# Genomic analysis uncovers laccase-coding genes and biosynthetic gene clusters encoding antimicrobial compounds in laccase-producing *Acinetobacter baumannii*

**DOI:** 10.1038/s41598-022-16122-5

**Published:** 2022-07-13

**Authors:** Renuka Pooalai, Supat Khongfak, Udomluk Leungtongkam, Rapee Thummeepak, Duangkamol Kunthalert, Sutthirat Sitthisak

**Affiliations:** 1 Department of Microbiology and Parasitology, Faculty of Medical Science, Naresuan University, Phitsanulok, Thailand; 2 Centre of Excellence in Medical Biotechnology, Faculty of Medical Science, Naresuan University, Phitsanulok, Thailand

**Keywords:** Biotechnology, Microbiology

## Abstract

Laccases are multicopper oxidase family enzymes that can oxidize various substrates. In this study, we isolated laccase-producing *Acinetobacter* spp. from the environment, and one isolate of laccase-producing *Acinetobacter baumannii,* designated NI-65, was identified. The NI-65 strain exhibited constitutive production of extracellular laccase in a crude extract using 2,6-dimethoxyphenol as a substrate when supplemented with 2 mM CuSO_4_. Whole-genome sequencing of the NI-65 strain revealed a genome size of 3.6 Mb with 3,471 protein-coding sequences. The phylogenetic analysis showed high similarity to the genome of *A. baumannii* NCIMB8209. Three laccase proteins, PcoA and CopA, that belong to bacterial CopA superfamilies, and LAC-AB, that belongs to the I-bacterial bilirubin oxidase superfamily, were identified. These proteins were encoded by three laccase-coding genes (*pcoA*, *copA*, and *lac*-AB). The *lac*-AB gene showed a sequence similar to that of polyphenol oxidase (PPO). Gene clusters encoding the catabolized compounds involved in the utilization of plant substances and secondary metabolite biosynthesis gene clusters encoding antimicrobial compounds were identified. This is the first report of whole-genome sequencing of laccase-producing *A. baumannii,* and the data from this study help to elucidate the genome of *A. baumannii* to facilitate its application in synthetic biology for enzyme production.

## Introduction

*Acinetobacter* spp. are Gram-negative bacteria that are widely found in soil and aquatic environments. To date, various *Acinetobacter* species have been studied for potential use in the bioremediation of industrial wastes. *Acinetobacter calcoaceticus* JC359 was found to decolorize azo dyes, and *Acinetobacter lwoffii* ENSG302 was applied to oxidize and detoxify methyl orange (MO), a carcinogen used in the textile industry^1,2^. Due to their ability to produce versatile enzymes, these bacteria have potential applications for use in biotechnology.

Laccases (EC 1.10.3.2) are copper-containing enzymes belonging to the superfamily of multicopper oxidases (MCOs) that can oxidize versatile substrates such as phenolic and nonphenolic compounds, lignin-related compounds, and highly fractious environmental pollutants^3^. Cupric ions (Cu^2+^) are crucial for forming metal-active sites in laccases and have been reported as laccase inducers, inducing the transcription of laccase genes and increasing enzymatic activity^4^. Due to their oxidizing ability, laccases have been identified for use in industrial and biotechnological applications^3,5,6^.

Laccases are produced by many organisms, most notably being studied in fungi. However, bacterial laccases, identified in some species such as *Bacillus amyloliquefaciens* and *Geobacillus yumthangensis,* have been less studied^7,8^. Bacterial laccase structures contain copper-binding motifs that are highly conserved and contain two or three cupredoxin-like domains. Laccases in bacteria are usually involved in metal homeostasis/oxidation, sporulation, morphogenesis, and cell and spore pigmentation^9^. Based on a systematic sequence-based classification and analysis tool from the Laccase and Multicopper Oxidase Engineering Database (https://lcced.biocatnet.de), laccases and related multicopper oxidases are classified into 16 superfamilies^10^. Most bacterial laccases are copper resistance proteins (CopA), bilirubin oxidases, copper efflux proteins (CueO), and bacterial multicopper oxidases (MCO). Laccase-coding genes are found in *E. coli* (*pcoA* and *cueO*, formerly *yacK*) and *P. syringae* (*copA*)^9^. Among *Acinetobacter baumannii* strains, laccase-coding genes such as *pcoA* and *copA*, which are structurally homologous to multicopper oxidases, were investigated, and it was found that these proteins are important for copper resistance^11^. However, very little is known about laccase-producing strains in *Acinetobacter* spp. To date, there is a lack of data involving the whole genome analysis of laccase-producing bacteria. Genomic information of laccase-producing bacteria is needed for understanding and applying these bacteria in industrial settings. DNA sequencing technologies play important roles in synthetic biology, which include improving microbial strain development for existing and novel bioproduct screening, discovering genes and pathways, and optimizing and understanding metabolic engineering of large-scale manufacturing^12^. Hence, in this study, we aimed to isolate laccase-producing *Acinetobacter* species, and to identify and perform genomic analysis of the laccase-coding genes and secondary metabolite biosynthesis genes of these isolates.

## Results

### Isolation and characterization of *Acinetobacter* spp.

From a total of 25 water samples and 28 soil samples, 17 *Acinetobacter* spp. were confirmed by a biochemical test and the detection of 16S rRNA and *rpo*B genes. Twelve isolates were from water, and five isolates were from soil. Species identification of all *Acinetobacter* spp. was performed by DNA sequencing of the *rpo*B gene. We found ten isolates that we identified as *A. baumannii,* of which five isolates were positive for the *bla*_OXA-51_ gene. Three isolates (17.65%) were assigned to *A. nosocomialis*. In addition, four isolates were identified as *A. soli*, *A. junii*, *A. pittii,* and *A. seifertii* (Supplementary Table S1).

### Production of laccase enzyme in *Acinetobacter baumannii* and antimicrobial susceptibility testing

**Figure 1:**
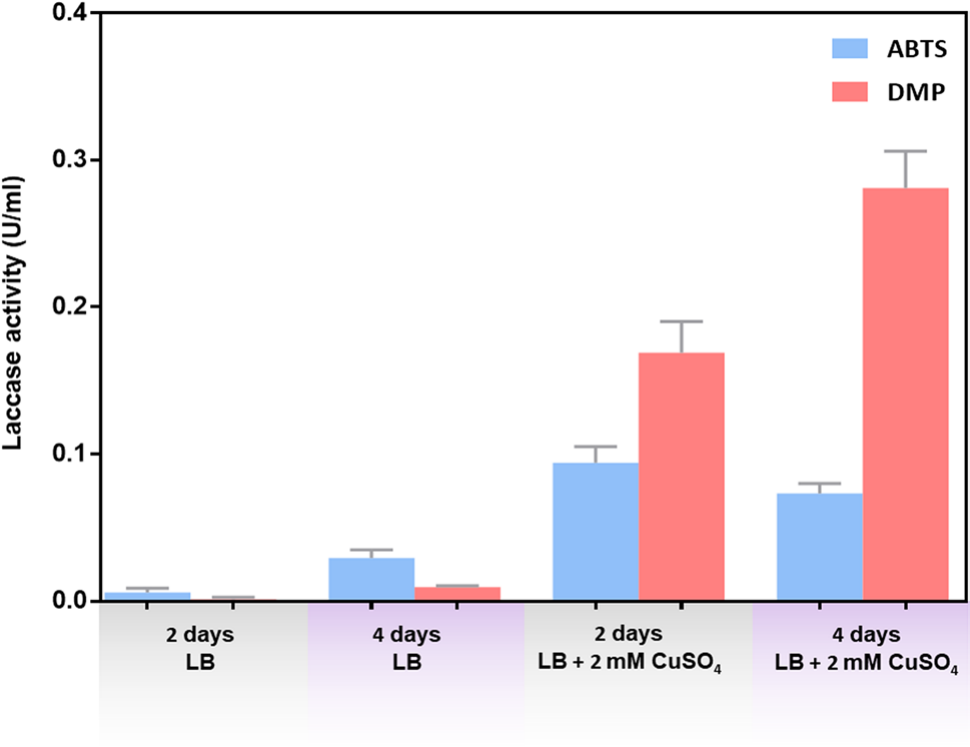
Effect of CuSO_4_ and incubation time on laccase production. The bacteria were grown in Luria–Bertani (LB) broth with and without 2 mM CuSO_4_ and incubated at room temperature for 2 and 4 days. The crude laccase activity from NI-65 was determined using ABTS (blue bar) and 2,6-DMP (red bar) as substrates. Values are means ± standard deviations of triplicate assays.

All *Acinetobacter* spp. were investigated for the production of laccase enzyme using a nutrient agar plate containing 0.02% guaiacol. Using the guaiacol assay, we found only one isolate (NI-65) that could produce the enzyme laccase, and this strain was identified as *A. baumannii* based on *rpo*B sequence analysis. The antibiotic susceptibility study of the NI-65 strain showed that it is sensitive to 14 antibiotics (Supplementary Table S2). Enzyme activity was determined from the crude extract using ABTS and 2,6-DMP as substrates. Laccase activity in the presence of 2 mM copper and the 2,6-DMP substrate was observed with an enzymatic activity of 0.3 U/mL after 4 days of incubation. We found no increase in induction when the copper concentration was increased. The low laccase activity was determined using ABTS as the substrate (Fig. 1 and Supplementary Table S3).

### Whole-genome analysis of NI-65

**Figure 2:**
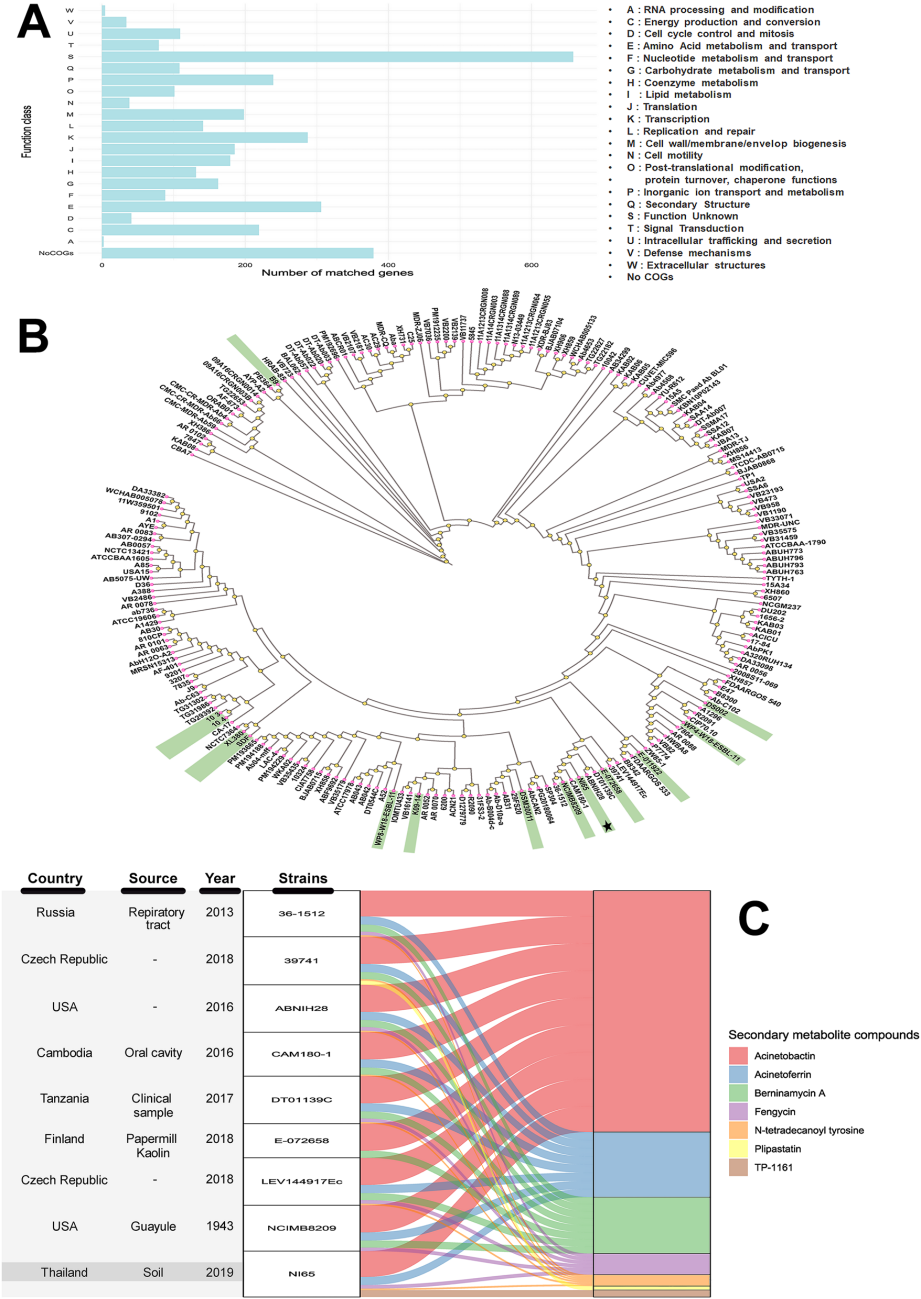
Genomic analysis of a draft assembled genome of NI-65. (**A**) Bar graph of COG functional classification of coding sequences. (**B**) Phylogenomic tree inferred from a draft genome of NI65 and 217 *A. baumannii* genomes deposited in the NCBI database. The position of NI-65 in the phylogenomic tree is marked with a black star. The *A. baumannii* isolates from the environment are highlighted in green. (**C**) An alluvial chart of secondary metabolite biosynthesis gene clusters identified in NI-65 and its closely related genomes by using antiSMASH version 5.1.2. (**A**) and (**C**) were illustrated using R studio version 1.4.1717. (**B**) was visualized and edited with iTOL (available at https://itol.embl.de/).

Sequencing of the NI-65 genome revealed a genome size of 3,666,760 bp with 39% GC content. A total of 3,471 predicted protein-coding and 64 tRNA genes were annotated in the NI-65 genome (Table 1). For COG assignments, the coding sequences identified in the genome were annotated and classified into different groups with 20 distribution classes, as presented in Fig. 2A. Intrinsic antibiotic resistance genes, including *bla*_OXA-51_ and *bla*_ADC-25_ which encode oxacillinases and cephalosporinases, respectively, were identified in the genome. One complete prophage was present in the genome. We also found virulence genes known to be involved in secretion systems (Type III and Type IV), adhesin, C5a peptidase, capsule formation, biofilm formation (*csuE, gacS, csuCD, ompA,* and *bfmS*), and toxins (cytolysin, cereulide, and neurotoxin A) (Table 1 and Supplementary Table S4). Furthermore, iron acquisition (*zur, sc1,* and *entE*) and copper resistance genes (*cueR, pcoAB, copRS,* and *oprC*) were also detected in the genome (Table 1). Phylogenetic analysis was performed using the core genome of NI-65 and 217 *A. baumannii* strains deposited in the NCBI database (Supplementary Table S5). As shown in Fig. 2B, a phylogenetic analysis revealed that NI-65 presented in the same cluster with the *A. baumannii* strains NCIMB8209 (98.57% ANI), CAM180-1 (98.53% ANI), 36-1512 (98.49% ANI), ABNIH28 (98.23% ANI), DT01139C (98.27% ANI), LEV144917EG (98.00% ANI), 39,741 (97.99% ANI), and E-072658 (97.73% ANI). Using the antiSMASH database, secondary metabolite biosynthetic gene clusters were predicted from the genomes of NI-65 and other closely related strains. The predicted secondary metabolite gene clusters in NI-65 were acinetobactin, acinetoferrin, fengycin, N-tetradecanoyl tyrosine, and TP-1161 (Fig. 2C and Supplementary Table S6).

**Table 1:** General features of *A. baumannii* NI-65 genome.

Features	*A. baumannii* NI-65
Length in bp	3,666,760
GC content (%)	39
Number of contigs	114
Number of genes	3540
Number of protein-coding genes	3471
Number of tRNA	64
Antimicrobial resistance genes	*bla*_OXA-51_, *bla*_ADC-25_
Virulence genes: biofilm formation	*csuE, gacS, csuCD, ompA, bfmS*
Virulence genes: secretion system	*pil*A*,* pilE/pilA, VPA0450, sseB, pieB/lirD, lem14, icsB, *rvhB6d, mavC, lpg2527, ankF/legA14/ceg31, pilQ, icmL/dotI, CBU_1665, virB5*
Virulence genes: capsule	*neuC, bexA, kpsM, cpsI*
Iron acquisition genes	*zur, sc1, entE*
Copper resistance genes	*cueR, pcoAB, copRS, oprC*
Plasmid replicon typing (GR)	Not found
MLST (pasture scheme)	ST490
Number of prophages (complete/incomplete)	3(1/2)
Bio project/Accession number	JAERPP000000000; BioSample SAMN17319840

**Figure 3:**
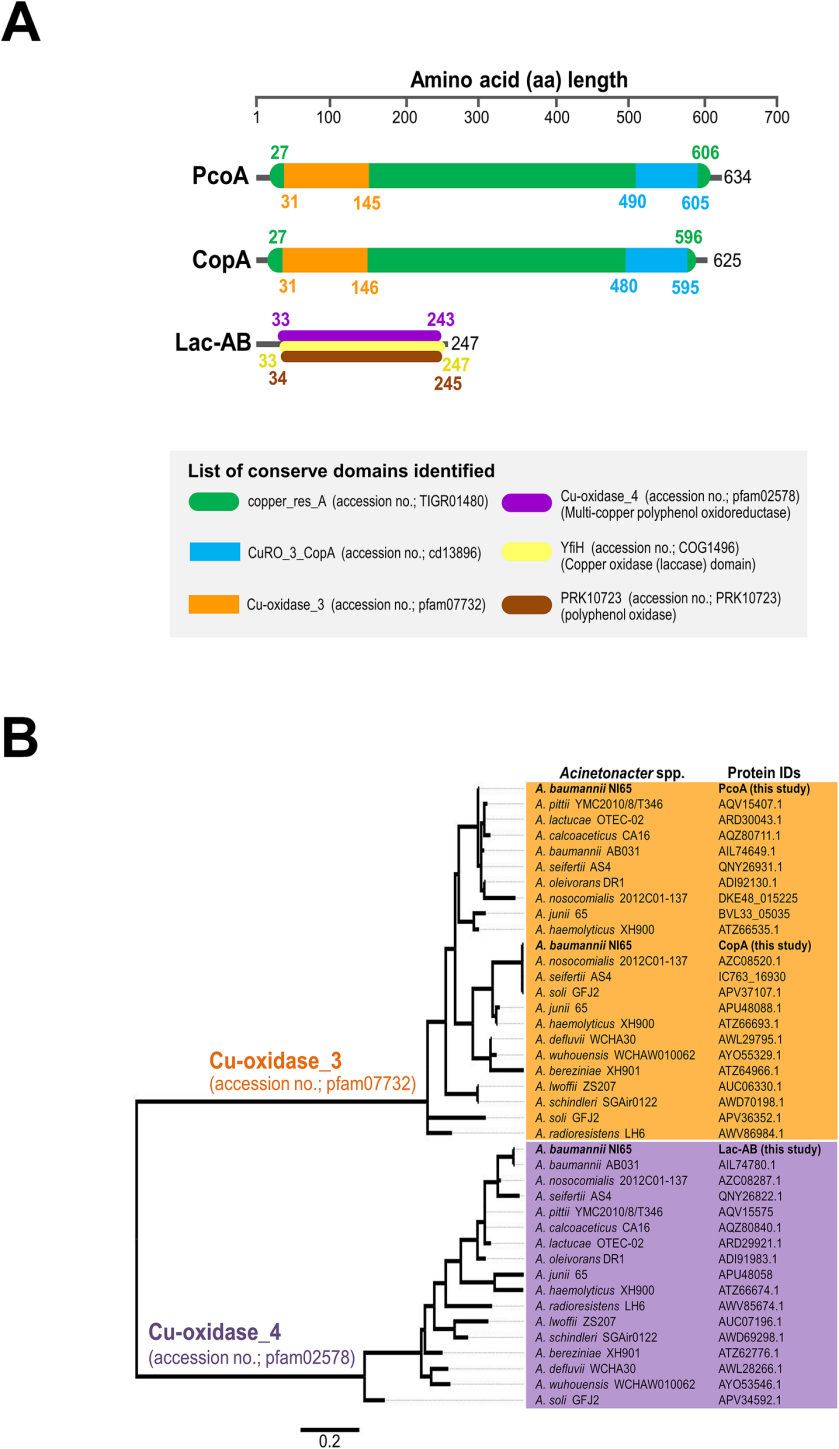
Conserved domains and phylogenetic tree analysis of three laccase proteins identified in the genome of NI-65. (**A**) The domain organization of three laccase proteins are PcoA, CopA, and Lac-AB. Each domain represented Cu-oxidase_3; Multicopper oxidase, CuRO_3_CopA; copper_res_A, the third cupredoxin domain of CopA copper resistance protein family; copper-resistance protein, CopA family, Cu-oxidase_4; Multicopper polyphenol oxidoreductase laccase, YfiH, copper oxidase domain, and polyphenol oxidase domain. (**B**) Phylogeny inferred from amino acid sequences of laccase coding-proteins containing Cu-oxidase_3 and Cu-oxidase_4 that were detected in the genomes of *Acinetobacter* spp. deposited at the NCBI database.

An analysis of the laccase proteins presented in the genome of NI-65 with sequences from the laccase and multicopper oxidase database (https://lcced.biocatnet.de/sequence-browser) revealed that two proteins, PcoA (NI65_02435) and CopA (NI65_01174), belonged to 100% identity with H-bacterial CopA superfamilies (Supplementary Table S7). A third laccase-coding protein (NI65_00511), belonging to the I-bacterial bilirubin oxidase superfamily (30% identity), was also detected. The gene coding for this protein was designated as the *lac*-AB gene. The domain organization of the three laccase-coding proteins is shown in Fig. 3A. The conserved domains identified in CopA and PcoA were Cu oxidase_3 (multicopper oxidase), CuRO_3_CopA (cupredoxin domain), and the copper-resistance domain. The conserved domains of the other laccase protein, LAC-AB (NI65_00511), were Cu-oxidase_4, the copper oxidase domain, and the polyphenol oxidase domain. The *lac*-AB gene (NI65_00511) was found in the genome of 217 *A. baumannii* strains retrieved from the NCBI database (Supplementary Table S8) with 90–100% identity, except for the VB31459 strain, which lacks this gene. A phylogenetic analysis of three laccase coding proteins found in *Acinetobacter* spp. showed that CopA and PcoA are in the same cluster as cu-oxidase_3 (Fig. 3B). Lac-AB was closely related to *Acinetobacter nosocomialis* and *Acinetobacter seifertii* (Fig. 3B). Among *Acinetobacter* spp., the *lac-*AB gene was detected with a 70–89% identity, and the conserved domain, Cu-oxidase_4, was detected in all *Acinetobacter* spp. (Fig. 2C and Supplementary Table S9).

## Discussion

*Acinetobacter* spp. are ubiquitous and can be obtained from water, soil, and human skin. Most of the studies of *Acinetobacter* spp. have been focused on clinically important aspects, such as the prevalence of antibiotic resistance. Recently, *Acinetobacter* spp. have attracted attention in environmental and biotechnological applications. Some strains of this genus are known to oxidize aromatic organic pollutants^1,2,12^. In this study, we isolated 12 strains of *Acinetobacter* from water and five strains from soil. They were identified as *A. baumannii, A. nosocomialis, A. soli, A. junii, A. pittii* and *A. seifertii.* We investigated the production of laccase enzymes from *Acinetobacter* spp. isolated using the guaiacol plate assay. We found only one isolate (NI-65) that produced laccase with the ability to  oxidize guaiacol, and this strain was identified as *A. baumannii*. This strain was selected to examine the laccase activity, and whole genome analysis was completed for this laccase-producing bacterium.

Previous reports of laccase-producing bacteria found that their enzymes can oxidize a broad range of substrates, such as phenol, pyrocatechol, syringaldazine, ABTS and guaiacol^13^. Kumar et al.^12^ isolated *Acinetobacter pittii* from olive mill wastewater (OMWW) based on its ability to biodegrade phenols and gallic acid. Using the ABTS substrate supplemented with CuSO_4_, this isolate was found to produce extracellular laccase with 1.5 U/mL activity. Notably, our study showed low laccase activity using 2,6-DMP substrates and ABTS supplemented with an optimal concentration of 2 mM CuSO_4_ (Fig. 1). Naturally, bacteria produce low concentrations of laccases, but the synthesis of the enzyme can be increased by adding inducers, such as CuSO_4_^3^. However, the optimal Cu concentration for the highest laccase production previously reported ranged from 0.5 to 2 mM^12,14^. Enzyme production and bacterial growth were inhibited under higher concentrations of Cu^2+^^14^.

Strain NI-65 was isolated from community forest soil. Although high resistance rates of *A. baumannii* environmental isolates against most antibiotics has been reported^15^, NI-65 was determined to be an antibiotic-sensitive strain, and only two intrinsic antibiotic resistance genes (*bla*_OXA-51_ and *bla*_ADC-25_) were identified. Biofilm formation and iron acquisition genes, which have been shown to be involved in the pathogenesis of most clinical *A. baumannii* isolates worldwide, were identified in NI-65. MCOs consist of different protein domains, which was the basis of their classification into 16 superfamilies^10^. An analysis of the laccase proteins of NI-65 found that CopA (NI65 01174) and PcoA (NI65_02435) belong to the bacterial CopA superfamily. Previously, CopA and PcoA were identified in *A. baumannii*, and their functions were deemed important to copper tolerance. The intrinsic *pcoA* gene was detected in all the isolates of *A. baumannii* tested, while *copA*, the acquired copper-tolerant gene, was detected in 20.64% of copper-tolerant *A. baumannii* isolates^11^. Our study also found that another laccase-coding gene (NI65_00511), the *lac*-AB gene, encoded polyphenol oxidase (PPO). In silico analysis revealed that the polyphenol oxidase gene was intrinsic and found in all 217 *A. baumannii* strains in the NCBI database (Supplementary Table S8). PPO has been characterized in the marine bacterium *Marinomonas mediterranea*, which exhibits laccase activity by the oxidation of the 2,6-DMP substrate^16^. In line with our study, we found that the production of extracellular laccase from NI-65 showed laccase activity using the 2,6-DMP substrate. Phylogenetic analysis of NI-65 and 217 *A. baumannii* strains revealed that NI-65 presented in a cluster with eight *A. baumannii* strains, albeit to separate clonal lineages (Fig. 2A,B). The highest similarity was found in *A. baumannii* strain NCIMB8209, isolated in 1943 from the aerobic degradation (retting) of desert guayule shrubs. This indicates that NI-65 shares a common ancestor with NCIMB8209.

A genomic analysis of NI-65 highlights many gene clusters that may be applied in synthetic biology to determine the compound that is hidden in the genome. The WGS analysis of NCIMB8209 showed a genome of 27 gene clusters encoding many catabolized compounds involved in the utilization of a large variety of plant substances^17^. These catabolic locus gene clusters were also detected in the NI-65 strain (Supplementary Table S10). These data imply that NI-65 has the ability to degrade and utilize plant substances. The presence of secondary metabolite biosynthetic genes was also investigated in the NI-65 strain using antiSMASH. Biosynthetic gene clusters encoding antimicrobial compounds such as fengycin (antifungal), TP-1161 (antibiotic), and N-tetradecanoyl tyrosine (antibiotic) were detected in NI-65 by the antiSMASH program. Fengycins are lipopeptides produced by *Bacillus* spp. that display potent antifungal activity against plant pathogens and multidrug-resistant foodborne pathogens^18,19^. This compound has great potential for application as a biocontrol agent in the agriculture and food industries. In addition, TP-1161, a thiopeptide antibiotic produced by marine *Nocardiopsis* species which exhibits antibiotic activity against a broad spectrum of multidrug-resistant Gram-positive bacteria and multidrug-resistant *Candida albicans,* has been identified in the genome of the NI-65 strain^20^. The last gene cluster identified in the NI-65 strain was N-tetradecanoyl tyrosine, which is a long-chain N-acyl amino acid isolated from soil bacteria with antibacterial activity^21^.

In conclusion, this research described the isolation, characterization, and draft genome sequencing of the *A. baumannii* NI-65 strain. This strain was isolated from soil with a drug-susceptible phenotype. The NI-65 strain shows laccase activity for the oxidation of guaiacol and 2,6-DMP. Genomic analysis shows that this strain possesses three laccase-coding proteins. CopA and PcoA function in copper tolerance, and LAC-AB has a sequence similar to PPO. Genomic analysis of the NI-65 strain revealed a metabolic pathway involved in the utilization of plant substances and contained biosynthetic gene clusters encoding compounds with antimicrobial activity. The genomic data will facilitate the application of the NI-65 strain in industrial production to improve strain development and optimize metabolic engineering for large-scale manufacturing in the future.

## Methods

### Isolation of laccase-producing *Acinetobacter* spp.

Soil and water samples were collected from Phitsanulok Province, Thailand. Five grams of collected soil was suspended in 50 mL of distilled water and mixed through a vortex. The aqueous phase was collected after centrifugation at 3000× *g* for 10 min at room temperature. Tenfold serial dilutions (through 10^−2^) of the supernatant were prepared in distilled water, and each dilution was spread onto Leeds Acinetobacter Medium (LAM). Water samples (50 mL) were collected, and tenfold serial dilutions (through-10^−2^) of the sample were prepared in distilled water, with each dilution spread onto the LAM. The agar plates were incubated at 30 °C for 24–48 h. The identification and characterization of *Acinetobacter* spp. were performed using the following tests: Gram staining, catalase, oxidase, triple sugar iron agar, oxidation fermentation, citrate, urea, ornithine decarboxylase, phenol red L-arabinose, and phenol red L-xylose broth. All positive isolates were further characterized using PCR amplification of the 16S rRNA, *rpo*B, and *bla*_OXA-51_ genes, and antimicrobial susceptibility testing was performed as previously described^11^. The protocol was approved by the Naresuan University Institutional Biosafety Committee (NUIBC) (No. NUIBC MI59-07-31).

### Screening of bacterial laccase and detection of laccase enzyme from the crude extract

All *Acinetobacter* spp. isolates were investigated for the presence of laccase activity using a nutrient agar plate containing 0.02% guaiacol as described by Umar et al., with some modification^22^. Guaiacol plate-test screening is an easy and cheap method for laccase screening, and the presence of a brown color around the margins of the colonies indicated the production of laccase (Supplementary Fig. S1). The crude extract was prepared from a strain that produced laccase, as determined by the guaiacol assay. Two substrates, 2,2′-azino-bis-(3-ethylbenzothiazoline-6-sulfonic acid) (ABTS) and 2,6-dimethoxyphenol (2,6-DMP), were used to characterize the unit activity of laccase. The bacteria were grown in Luria–Bertani (LB) broth with and without 2 mM CuSO_4_ and incubated at room temperature for 2 and 4 days. Measuring the laccase activity using the ABTS substrate was a method that we adapted from Childs and Bardsley^23^ that resulted in a final concentration of 2 mM ABTS in a 0.1 M sodium acetate buffer (pH 4.0) followed by a 5 min incubation at room temperature. The increased absorbance was measured at 420 nm (ε420 = 36,000 M^−1^ cm^−1^). The 2,6-DMP solution, formulated according to Heinzkill et al.^24^, contained 5 mM 2,6-DMP in 0.1 M sodium acetate buffer (pH 4.0) and was incubated at room temperature for 10 min. The increased absorbance was measured at 469 nm (ε469 = 27,500 M^−1^ cm^−1^). One unit of enzyme activity was defined as the amount of enzyme needed to oxidize 1 μmol ABTS or 2,6-DMP in 1 min.

### Identification of *Acinetobacter* spp. and whole-genome sequencing (WGS) of laccase-producing *Acinetobacter* spp.

The molecular identification of all bacterial isolates was performed by DNA sequencing of the *rpo*B gene. The PCR products of *rpo*B from all isolates were purified and sequenced. BLAST was used to compare the sequences against the GenBank database (https://blast.ncbi.nlm.nih.gov/BLAST). BLASTn was conducted with an e-value cutoff of 1e-10, minimum 80% identity, and 95% query coverage. The laccase-producing strain was selected for whole-genome analysis. Genomic DNA was extracted and sequenced by the Illumina MiSeq platform (250 bp paired-end). Reads were trimmed and assembled by using Sickle v1.3337 and SPAdes genome assemblers v3.6.038 with default settings. After assembling contigs, annotation was conducted with the RAST pipeline using default parameters.

### Data analysis

Sequences from the laccase and multicopper oxidase database were used as queries for the BLASTp search. Laccase proteins present in the genome of NI-65 were identified using pair BLASTp. The complete genome sequences of 216 *A. baumannii* and 1 draft genome were retrieved from the NCBI database for phylogenomic analysis (Supplementary Table S5). Clusters of orthologous genes (COGs) were identified using the eggNOG-Mapper web server^25^. Then, the bar graph was illustrated using R studio version 1.4.1717. A core-genome SNP-based phylogeny of NI-65 and an additional 217 *A. baumannii* genomes was constructed using CSI Phylogeny^26^, and the phylogenetic tree was visualized and edited with Interactive Tree Of Life (iTOL) (available at https://itol.embl.de/). FastANI v1.3 was used to find a similar genome to NI-65 based on the average nucleotide identity (ANI) percentage^27^. Secondary metabolite biosynthesis gene clusters were identified using antiSMASH (Version 5.1.2)^28^. The results were illustrated using R studio version 1.4.1717. Catabolic loci were detected using BLASTn (with coverage and identity thresholds of 70% and an E-value cutoff of 0.001) and BLASTp (with an E-value of 0.001, a minimal identity value of 30%, and a coverage of > 75%) based on the Galaxy framework. The genomes of NCIMB8209 and DSM30011 were used as DNA query sequences. ABRicate v 1.0.1 was used to screen the antimicrobial resistance genes^29^. The presence of virulence genes in the genome of NI-65 was analyzed on VFanalyzer (http://www.mgc.ac.cn/cgi-bin/VFs/v5/main.cgi?func=VFanalyzer). Conserved domains from three laccase proteins identified in the genome of NI-65 were identified using BLASTp.

## Supplementary Information

Supplementary Information.

## Data Availability

Datasets for this research are included in the supplemental figures and tables. The whole genome has been deposited at DDBJ/ENA/GenBank under the accession number JAERPP000000000; BioSample SAMN17319840. The sequence can be retrieved from https://www.ncbi.nlm.nih.gov/nuccore/.

## Abbreviations

2, 6-DMP: 2, 6-Dimethoxyphenol
ABTS: 2, 2′-Azino-bis 3-ethylbenzothiazoline-6-sulphonic acid
ANI: Average nucleotide identity
BLASTn: Basic local alignment search tool for nucleotide
BLASTp: Basic local alignment search tool for protein
COGs: Clusters of Orthologous Genes
NCBI: National Center for Biotechnology Information
PPO: Polyphenol oxidase
U/mL: Enzyme activity unit/mL
WGS: Whole genome sequence
